# Early and late scanning electron microscopy findings in diabetic kidney disease

**DOI:** 10.1038/s41598-018-23244-2

**Published:** 2018-03-20

**Authors:** Sara Conti, Norberto Perico, Rubina Novelli, Camillo Carrara, Ariela Benigni, Giuseppe Remuzzi

**Affiliations:** 1 IRCCS - Istituto di Ricerche Farmacologiche Mario Negri, Bergamo, Italy; 2 Unit of Nephrology and Dialysis, Azienda Socio-Sanitaria Territoriale Papa Giovanni XXIII, Bergamo, Italy; 3 Department of Biomedical and Clinical Sciences ‘L. Sacco’, University of Milan, Milan, Italy

## Abstract

Diabetic nephropathy (DN), the single strongest predictor of mortality in patients with type 2 diabetes, is characterized by initial glomerular hyperfiltration with subsequent progressive renal function loss with or without albuminuria, greatly accelerated with the onset of overt proteinuria. Experimental and clinical studies have convincingly shown that early interventions retard disease progression, while treatment if started late in the disease course seldom modifies the slope of GFR decline. Here we assessed whether the negligible renoprotection afforded by drugs in patients with proteinuric DN could be due to loss of glomerular structural integrity, explored by scanning electron microscopy (SEM). In diabetic patients with early renal disease, glomerular structural integrity was largely preserved. At variance SEM documented that in the late stage of proteinuric DN, glomerular structure was subverted with nearly complete loss of podocytes and lobular transformation of the glomerular basement membrane. In these circumstances one can reasonably imply that any form of treatment, albeit personalized, is unlikely to reach a given cellular or molecular target. These findings should persuade physicians to start the putative renoprotective therapy soon after the diagnosis of diabetes or in an early phase of the disease before structural integrity of the glomerular filter is irreversibly compromised.

## Introduction

Type 2 diabetes is a public health concern and predictions regarding its future effects on human and social costs, as well as health economic impact are staggering^1,2^. Diabetic Kidney Disease (DKD), the most devastating complications of type 2 diabetes, is the single leading cause of end-stage renal disease (ESRD) in the industrialized world^3^.

The natural history of DKD in type 2 diabetes has been recently depicted as a sequence of stages characterized initially by normal or elevated (hyperfiltration) glomerular filtration rate followed by progressive renal function decline to end-stage renal disease^4–6^. Progressive renal function loss can be noted even in patients with normoalbuminuria as well as in those with microalbuminuria, but the onset of macroalbuminuria or overt proteinuria is invariably associated with accelerated rate of renal disease progression^4^.

Current strategies to prevent or slow DKD progression of type 2 diabetes focus on metabolic and blood pressure control, as well as on reducing protein trafficking through the glomerular capillary barrier, mainly through blockade of the renin-angiotensin system (RAS) with angiotensin-converting enzyme inhibitors (ACEi) and/or angiotensin II type 1 receptor blockers (ARBs)^7^. In a proteinuric rat model of streptozotocin-induced diabetes the administration of an ACE-inhibitor normalized the urinary protein excretion when treatment was started early in the course of the disease, but not at later time, when proteinuria was elevated^8^. The effect of ACE inhibition on proteinuria was associated with protection against the development of glomerular structural changes only early, but not later in the course of disease^8^. Consistent with these findings in the animal model, in the early 2000s large clinical trials in type 2 diabetic patients showed that RAS inhibition provided some degree of protection against renal disease progression in the course of proteinuric DKD, but early interventions (normoalbuminuria, BENEDICT^9^ or microalbuminuria, IRMA-2^10^) were more beneficial than late intervention (overt proteinuria, RENAAL^11^, IDNT^12^) in delaying ESRD^13^. Moreover, in the advanced nephropathy of RENAAL and IDNT trials consistent renoprotection was only achieved in those patients who reported a reduction in urinary protein excretion^11,12^. More recent clinical trials in type 2 diabetic patients with overt proteinuria have also largely failed to provide significant renoprotection independently of the novel treatments investigated^14^. Collectively, these observations highlight the variability of response to treatment during the course of the disease with negligible benefit in later stages of proteinuric DKD.

One of the principal obstacles to the development of improved therapeutic interventions remains our limited in depth understanding of the structural abnormalities at glomerular level in patients with proteinuric advanced DKD.

Thus, in the present study we sought to investigate whether failure to renoprotection by the available treatments in DKD with overt nephropathy could be related to loss of glomerular structural integrity. To this purpose, we took advantage of scanning electron microscopy (SEM), an imaging approach rarely used for patients^15^, which provides a global visualization of the actual three-dimensional appearance of the glomerular tuft surface and podocyte cytoarchitecture. SEM allows a more in depth information on glomerular injury than that already available in diabetic patients with a single cross-section at light as well as transmission electron microscopy (TEM)^16^.

## Results

In order to establish the reliability of our scanning electron microscopy (SEM) procedure, we first examined kidney specimens of healthy tissue obtained from non-diabetic controls undergoing nephrectomy for renal adenocarcinoma without histological evidence of glomerular disease. SEM analysis showed well preserved glomerular architecture with normal peripheral capillary loop ultrastructure (Supplementary Figure S1). The outer surfaces of glomerular capillaries were covered by highly branched podocytes displaying intact cell bodies and primary processes with well-organized interdigitating foot processes (Supplementary Figure S1, B and D).

To in depth characterize early and late renal structural injury in type 2 diabetes, we analyzed with SEM autopsy specimens from two patients with early DKD (normo- or micro-albuminuria), and compared the three-dimensional appearance of glomeruli with that of biopsy specimens from six patients with late type 2 DKD and overt proteinuria, all receiving RAS therapy as part of their current renoprotective treatment.

**Figure 1:**
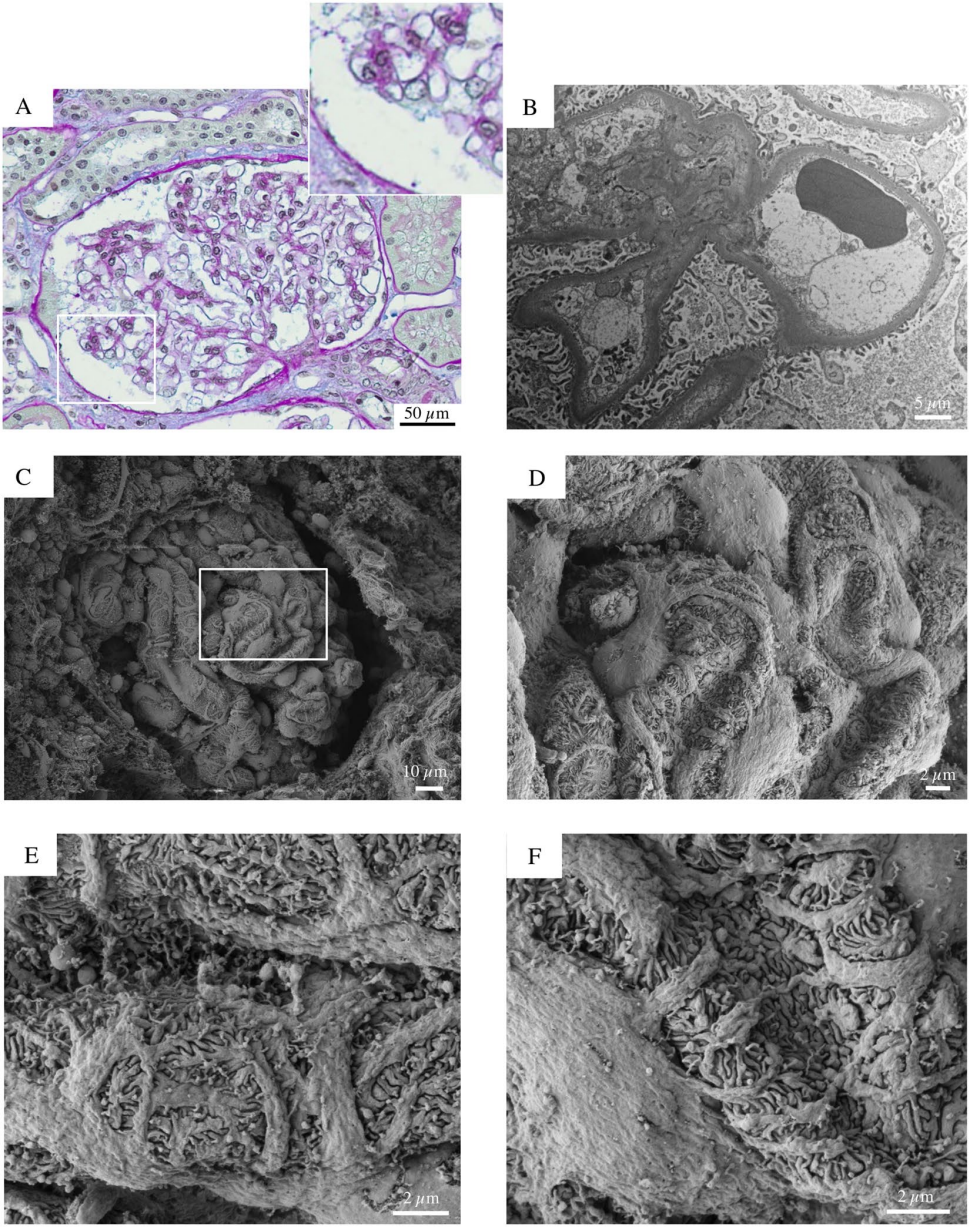
Glomerular histologic and ultrastructural findings from the normoalbuminuric diabetic patient. (**A**) A light microscopy image reveals a normocellular glomerulus with mild segmental mesangial expansion and patent capillary lumina. The surrounding tubular parenchyma doesn’t have any sign of injury (periodic acid-Schiff). (**B**) A transmission electron micrograph shows normal peripheral capillary loops covered by intact podocyte foot processes. (**C**) Glomerular scanning electron micrographs with the corresponding high magnification insets (**D**,**E** and **F**). (**C**) A well-preserved glomerulus without any evident signs of injury partially emerges from the renal cortex surface. (**D**,**E** and **F**) The urinary side of the capillary wall is covered by highly branched podocytes (**E**), which are morphologically intact and unaltered (**F**).

As shown in Fig. 1C–F, SEM examination of glomeruli from autopsy specimens of a 61-year-old woman with very early stage of DKD (normo-albuminuria) revealed well preserved outer surfaces of glomerular capillaries characterized by highly branched podocytes. In particular, the podocytes had intact cell bodies and primary processes, with a normal foot process interdigitating distribution pattern, completely covering the GBM surface. On average, glomerular size and podocyte body volume appeared moderately increased. These patterns are consistent with expected early stage DKD abnormalities, which are characterized by glomerular hypertrophy and a mild increase in mesangial matrix^17^.

**Figure 2:**
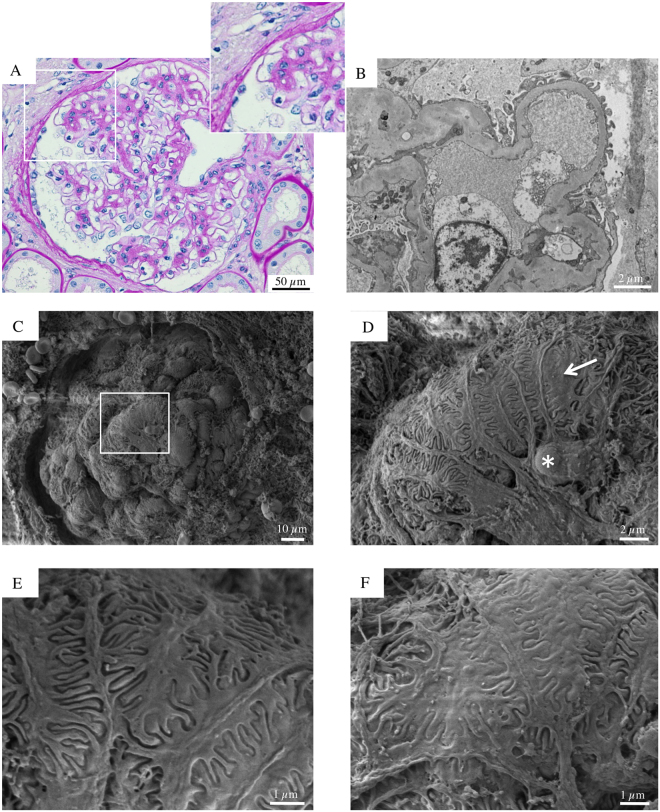
Glomerular histologic and ultrastructural findings from the microalbuminuric diabetic patient. (**A**) A light microscopy image reveals a glomerulus with vaguely nodular mesangial expansion and prominent capillary loops. The surrounding tubular parenchyma reveals initial mild tubular atrophy (periodic acid-Schiff). (**B**) A transmission electron micrograph shows peripheral glomerular capillaries with thickened GBM and segmental effacement of the overlying epithelial cells. The mesangial regions are expanded due to increased matrix. (**C**) Glomerular scanning electron micrograph with the corresponding high magnification insets (**D**,**E** and **F**). (**C**) A partially visible glomerular tuft appears moderately expanded. A podocyte seemingly hypertrophic with a stretched and flattened cell body and initial effacement of the interdigitating foot processes (**D** arrow, **E** and **F**). Occasional pseudocysts of podocyte cell bodies are seen (**D**, asterisk).

SEM evaluation provided more relevant findings in a 70-year-old man with microalbuminuric diabetic nephropathy. In this setting, the examination of the renal cortex surface suggested a glomerular adaptative growth, with a consequent mismatch between the expansion of the tuft and the hypertrophy of podocytes, which displayed stretched and flattened cell bodies (Fig. 2C–F). In addition, in this biopsy we observed three other findings that could represent important features of podocyte injury. First, the attenuated visceral epithelial cell cytoplasm, here and there, bulged with occasional pseudocysts (Fig. 2D and E, asterisk), representing sites of initial cells detachment from the GBM; second, the visceral epithelial cells interdigitating foot processes had diffuse effacement (Fig. 2D, arrow, and F) and; third, the proportion of the interdigitation area appeared reduced by shortening and degradation of some foot processes.

**Figure 3:**
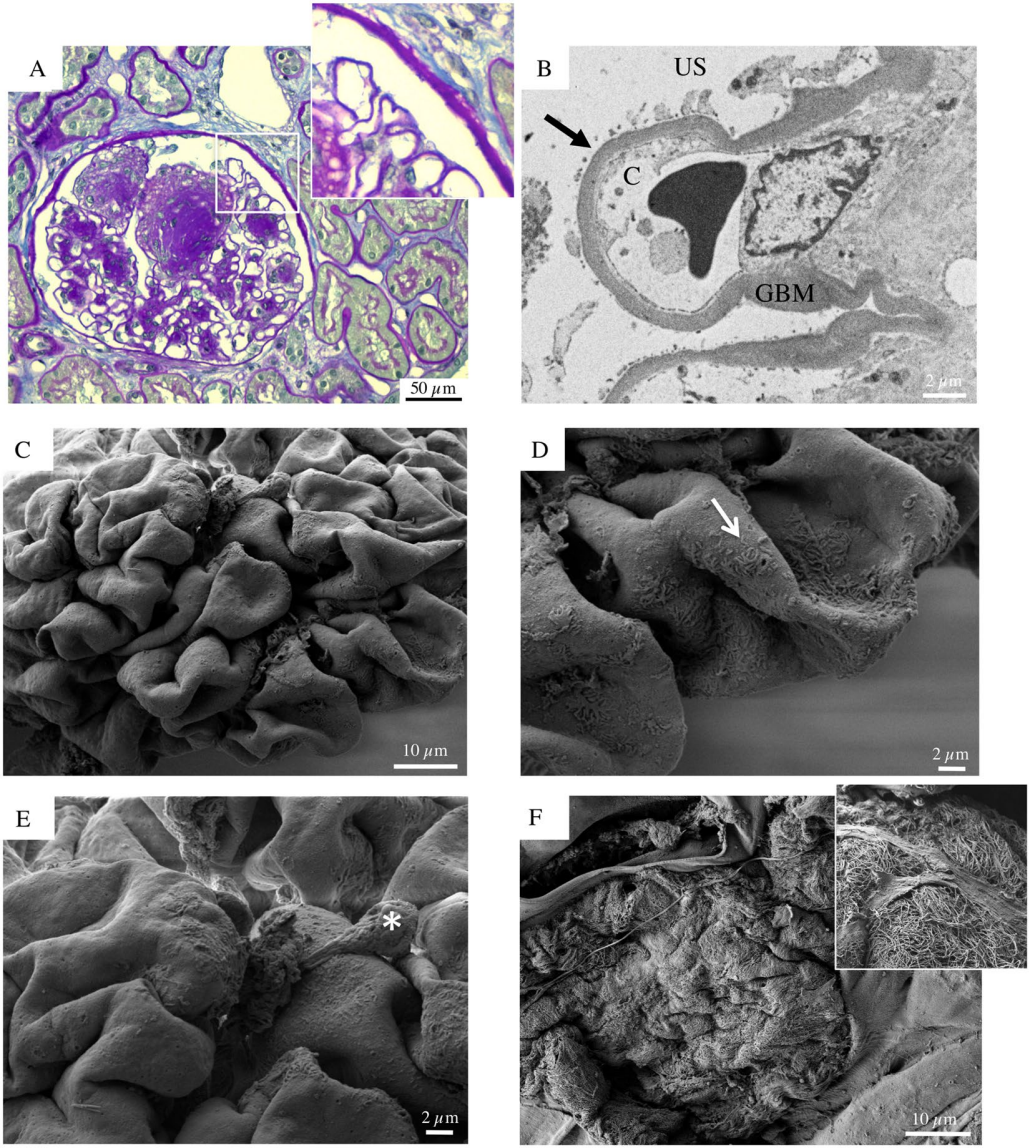
Glomerular histologic and ultrastructural findings in advanced DKD. Patient 2. (**A**) A light microscopy image shows an hypertrophic glomerulus with prominent nodular expansion of the mesangium due primarily to increased matrix (Kimmelstiel-Wilson nodules). Other features of these nodules are mesangiolysis and initial microaneurysm formation. The surrounding tubular parenchyma reveals somewhat diffuse mild interstitial fibrosis, associated with focal tubular atrophy (periodic acid-Schiff). (**B**) A transmission electron micrograph documents dramatic denudation of GBM (arrow). C, capillary lumen; GBM, glomerular basement membrane, US, urinary space. (**C**) Glomerular scanning electron micrographs, with the corresponding high magnification insets (**D** and **E**). A large, mostly acellular glomerulus, protrudes from the renal cortex surface. (**D**, arrow) Fragments of foot processes remained in very few areas. (**E**, asterisk) A lonely podocyte, with a vague bottle-shaped cellular body, appears to be almost nearly completely detached from the underlying GBM surface. (**F**) The surface of a sclerotic glomerulus with an extensively shattered and fragmented GBM. (**F**, inset) A tangle of elongated collagen fibrils has replaced the typical glomerular structures.

**Figure 4:**
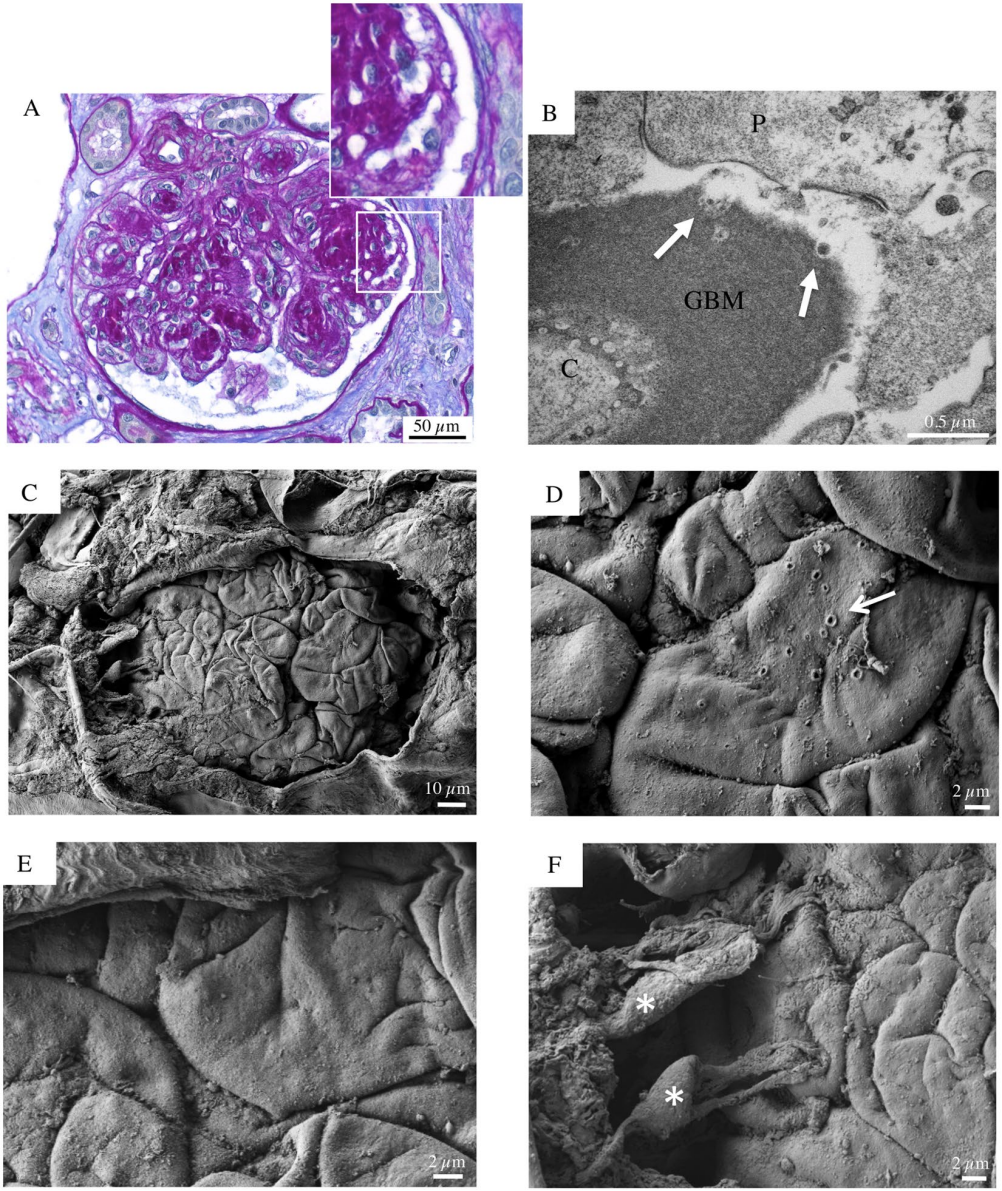
Glomerular histologic and ultrastructural findings in advanced DKD. Patient 3. (**A**) A light microscopy image shows a large glomerulus with nodular mesangial expansion due primarily to increased matrix (Kimmelstiel-Wilson nodules) associated with initial tuft-to-capsule adhesion. Occasional circulating leucocytes are seen within glomerular capillary lumina. The surrounding tubular parenchyma reveals somewhat diffuse mild interstitial fibrosis (periodic acid-Schiff). (**B**) A transmission electron micrograph demonstrates a denuded GBM with ‘crater-like’ formations (arrows). C capillary lumen; GBM, glomerular basement membrane; P, podocyte. (**C**) Glomerular scanning electron micrographs, with the corresponding high magnification insets (**D**,**E** and **F**). An acellular glomerulus, partly covered by a damaged Bowman’s capsule, shows denuded peripheral capillary loops characterized by irregular and convoluted folds (**E**). (**D**, arrow) Shallow “crater-like” formations of the GBM surface are noted. (**F**) A cluster of nearly completely detached podocytes (asterisks), exhibiting stretched end elongated primary processes, forms a bridge between the Bowman’s capsule and the GBM surface.

**Figure 5:**
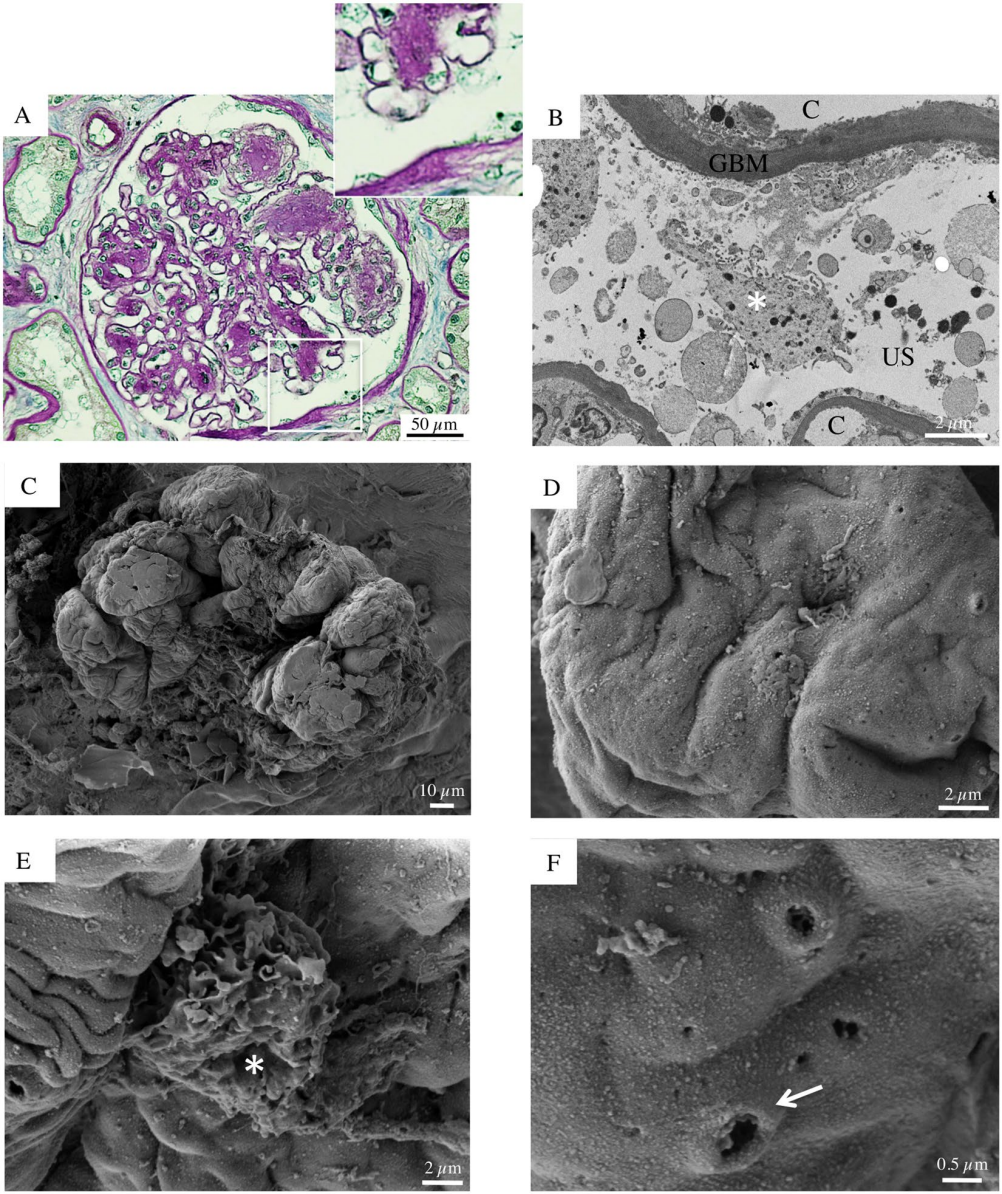
Glomerular histologic and ultrastructural findings in advanced DKD. Patient 1. (**A**) A light microscopy image shows an hypertrophic glomerulus with segmentally nodular mesangial expansion, due primarily to increased matrix (Kimmelstiel-Wilson nodules) and features of mesangiolysis. The surrounding tubular parenchyma reveals somewhat diffuse interstitial fibrosis associated with focal tubular atrophy (periodic acid-Schiff). (**B**) A Transmission electron image demonstrates podocyte microvillous transformation (asterisk). C, capillary lumen; GBM, glomerular basement membrane; US, urinary space. (**C**) Glomerular scanning electron micrographs, with the corresponding high magnification insets (**D**,**E** and **F**). (**C** and **D**) A large, mostly acellular glomerulus, exhibits “cauliflower-like” lobulations with loss of the typical cylindrical shape of glomerular capillaries. (**E**, asterisk) A lonely podocyte shows loss of most of its primary and secondary processes, severe degeneration of its cellular cytoarchitecture and microvillous transformation. (**F**, arrow) Higher magnification of the GBM surface reveals occasional cavities and tunnels.

**Figure 6:**
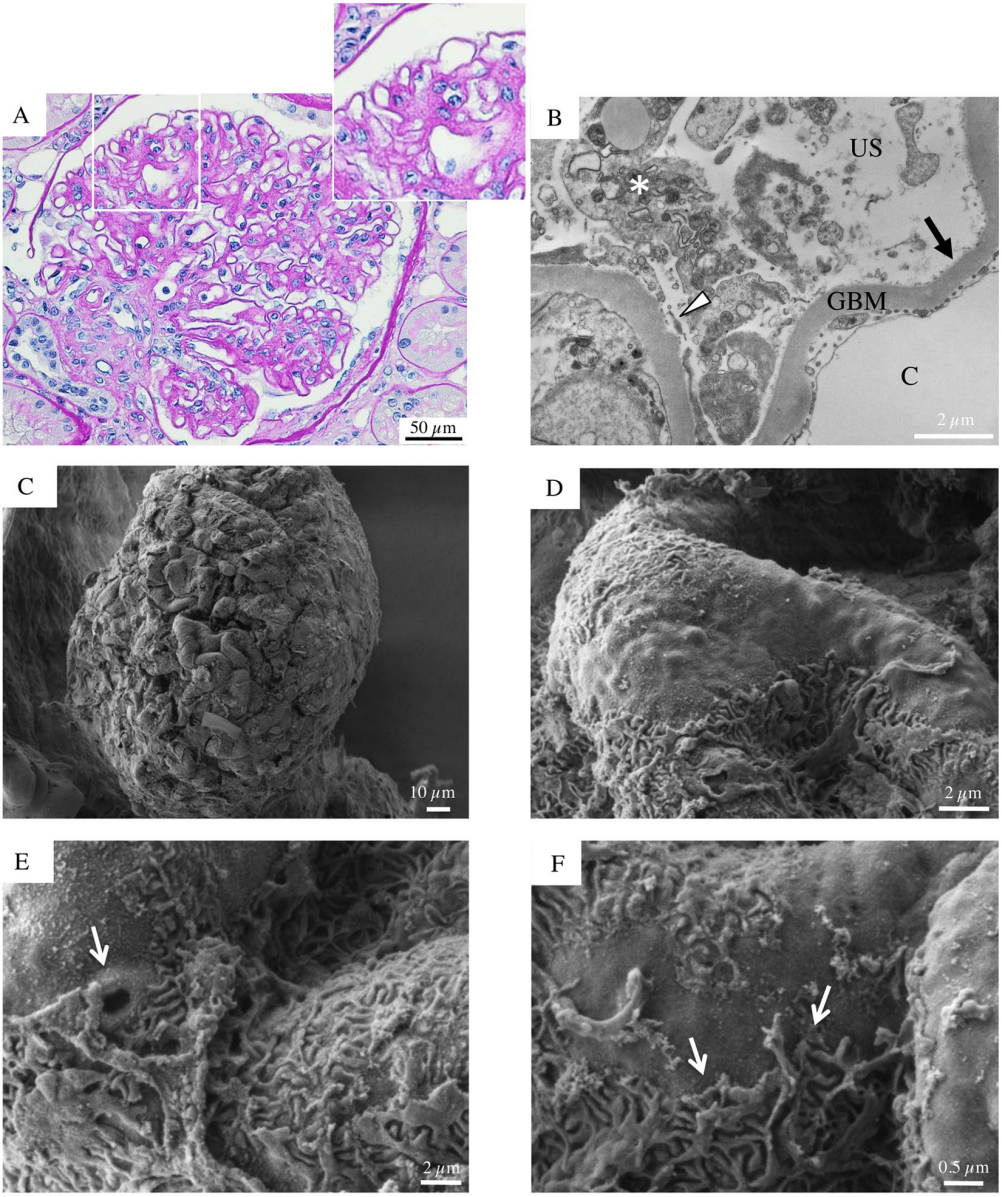
Glomerular histologic and ultrastructural findings in advanced DKD. Patient 4. (**A**) A light microscopy image shows a vaguely lobulated glomerulus with diffuse moderate mesangial expansion due primarily to increased matrix (periodic acid-Schiff). (**B**) A transmission electron micrograph reveals segmental denudation of the GBM (arrow) with detachment (arrowhead), lysis and fragmentation of the overlying podocyte (asterisk). (**C**) Glomerular scanning electron micrographs, with the corresponding high magnification insets (**D**,**E** and **F**). A large glomerulus protrudes from the renal cortex surface. (**D–F**) Areas of denuded GBM are frequently observed along the capillary loop surface, which is still partially covered by highly damaged foot processes (**F**, arrows). (**E**, arrow) Occasional cavities and tunnels are noted on the GBM surface.

**Figure 7:**
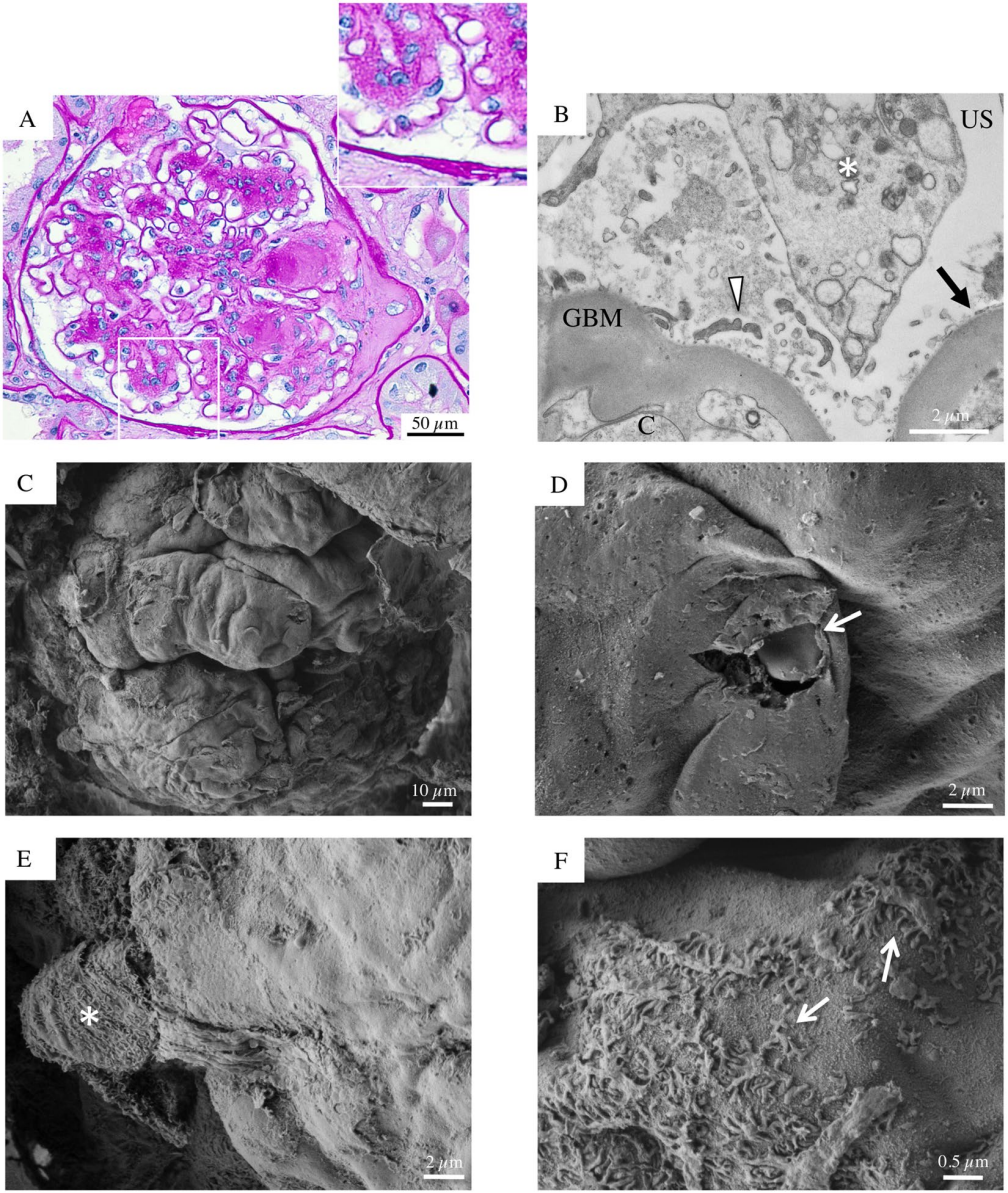
Glomerular histologic and ultrastructural findings in advanced DKD. Patient 5. (**A**) A light microscopy image shows a glomerulus with frequently nodular mesangial expansion, due primarily to increased matrix (Kimmelstiel-Wilson nodules), microaneurysm formation and obstruction of the glomerular outflow tract by segmental sclerosis at the glomerulo-tubular junction (periodic acid-Schiff). (**B**) A transmission electron micrograph documents extensively denuded GBM accompanied by degenerated podocyte bodies (asterisk) with isolated secondary processes detectable (arrowhead). (**C**) Glomerular scanning electron micrographs, with the corresponding high magnification insets (**D**,**E** and **F**). A mostly acellular glomerulus, partially visible from the Bowman’s capsule, displays naked subepithelial surface with a smooth appearance (**C** and **D**). (**D**, arrow) A blood cell protrudes through an accidentally broken glomerular capillary wall. (**E**, asterisk) A podocyte clearly shows a severe alteration of its structure and microvillous transformation. Sparse and damaged foot processes are detected in focal areas (**F**, arrows).

**Figure 8:**
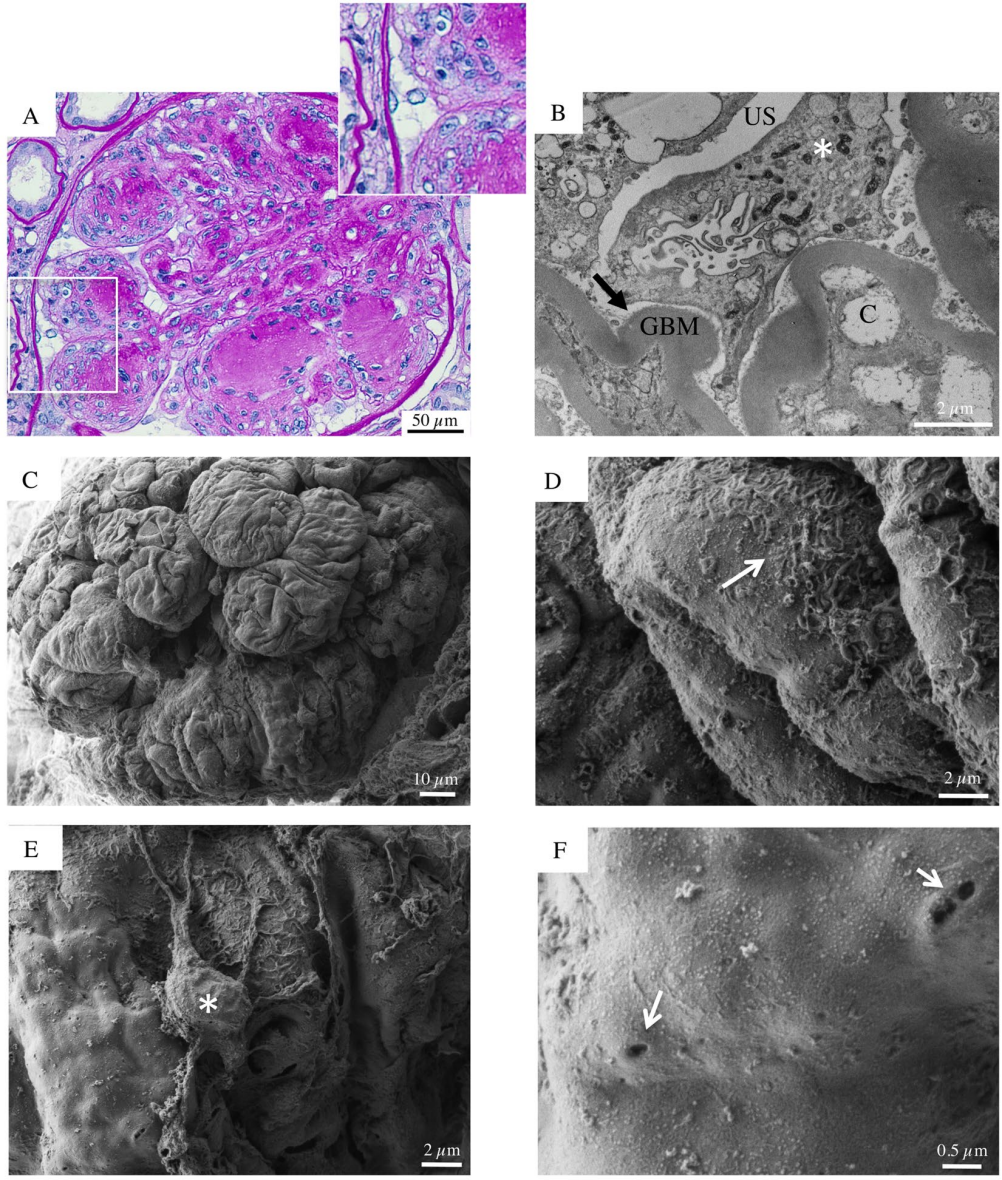
Glomerular histologic and ultrastructural findings in advanced DKD. Patient 6. (**A**) A light microscopy image shows a large glomerulus with severe nodular mesangial expansion due primarily to increased matrix (Kimmelstiel-Wilson nodules) associated with multiple tuft-to-capsule adhesions (periodic acid-Schiff). (**B**) Transmission electron micrograph reveals large regions of podocyte’s foot processes detachment from the GBM (arrow). In addition, podocytes exhibit features of microvillous transformation and cytoplasmic vacuolization (asterisk). (**C**) Scanning electron micrographs of a glomerulus from patient 6, with the corresponding high magnification insets (**D**,**E** and **F**). An acellular glomerulus partially emerges from the renal cortex surface (**C**). The GBM is almost completely denuded, and only focal areas with severely damaged foot processes are observed (**D**, arrows). (**E**, asterisk) A partly detached podocyte displays morphologically altered cellular body and severe alteration of its branching structure. (**F**) Cavities and tunnels are spotted along the GBM surface.

Biopsy specimens from six patients (aged 50 to 73) with DKD and overt proteinuria, despite therapy with ACEi and ARB, were also evaluated (Figs 3–8). In all patients, except one (Patient 4), light microscopy showed glomeruli with diffuse, frequently nodular, mesangial expansion, due primarily to increased mesangial matrix (Kimmelstiel-Wilson nodules) and often exhibited mesangiolysis, characterized by fraying of the interface between the mesangium and the capillary loops (Figs 3, 4, 5, 7, 8A and Supplementary Figure S2). The Bowman’s capsule was typically thickened. The tubular parenchyma had broad areas of interstitial fibrosis, tubular atrophy, associated with patchy interstitial inflammation. There was moderate to focally severe arteriolar hyalinosis. A surface examination of renal tissue by SEM consistently showed dramatically altered glomerular structures in these patients with advanced stage DKD. In particular, SEM revealed almost completely acellular glomeruli characterized by a naked subepithelial surface with a smooth appearance (Figs 3–5C and 7–8C). TEM examination confirmed that GBM was largely denuded (Figs 3–8B), and fragments of degenerated foot processes were frequently observed along the GBM (Figs 6B and 7B, white arrowheads), although these features were more intelligible with the 3D view available with SEM (Figs 3D, 6–7F and 8D). Only a very few partly detached podocytes were seen here and there with morphologically altered cellular bodies (Figs 3 and 5E, 4F, 7 and 8E asterisks), recalling microvillous transformation (Fig. 5B, asterisks) and occasionally forming bridges between the tuft and Bowman’s capsule (Fig. 4F, asterisks).

SEM images also showed that the external surface of GBM peripheral loops in these patients had irregular folds, already described in DKD as ‘cauliflower-like’ lobulations^18^ (Fig. 5C and D). Further magnification of the glomeruli showed occasional shallow ‘crater-like’ formations on the GBM surface, with a variable diameter of 20 to 350 nm, which are likely footprints of detached subepithelial immune complex (Figs 4B and D, arrows). We also observed other ultrastructural GBM defects different from ‘craters’, likely corresponding to ‘nephrotic tunnels and cavities’ previously detected by TEM studies in patients with nephrotic syndrome^19^ and in diabetic nephropathy with massive proteinuria^20^. The size and shape of these tunnels and cavities varied about 10 to 200 nm in diameter, and they were scattered diffusely along the GBM surface (Figs 5F, 6E and 8F, arrows).

Interestingly, in one of the biopsies, we fortuitously spotted a blood cell protruding through an accidentally broken glomerular capillary (Fig. 7D) indicating that, despite the massive structural damage clearly visible by SEM analysis, the glomerulus was likely still functional.

Renal biopsy from Patient 4 demonstrated slightly different findings compared to the other DKD patients with overt proteinuria. In general, the degree of glomerular injury was less severe. In particular, light microscopy showed a vaguely lobulated glomerulus with moderate mesangial expansion, but not clear Kimmelstiel-Wilson nodules or features of mesangiolysis (Fig. 6A). Both TEM and SEM revealed a segmental pattern of GBM denudation, with some regions of the capillary loop surface partially covered by highly damaged podocytes (Fig. 6B,C,D,E,F and Supplementary Figure S3). Few preserved areas of glomerular tuft were detectable by SEM (Supplementary Figure S3-C, arrowhead and S3-D), near denuded capillary loop surfaces (Supplementary Figure S3-C, arrow and S3-E), connected by a microvillous podocyte (Supplementary Figure S3-C, asterisk). Strikingly, in these rare preserved areas, SEM analysis showed the porous ultrastructure of glomerular filtration slits (Figure S3-F and S3-G, insets). The observation of glomerular filtration pores, recently identified by our group as main actors in the glomerular filtration permselectivity^15^ suggested that the filtration barrier was structurally intact in some areas of the glomerular capillary tuft. This finding further ruled out that the nearby observed denudated areas on the capillary loop surfaces may represent an artificial damage due to SEM sample processing.

Finally, Fig. 3F illustrates the most advanced phase of glomerular obsolescence in a case of overt DKD. The GBM is extensively shattered and fragmented, whereas the underlying surface exhibited a tangle of elongated fibrils which had replaced the typical glomerular structures, creating a fibrillar collagen network.

## Discussion

In this study we observed, by SEM three-dimensional analysis, a complete loss of glomerular structural integrity in patients with type 2 diabetes and overt nephropathy despite treatment with RAS inhibitors/blockers. At variance, SEM documented a largely preserved glomerular structure in diabetic patients with early stage of renal disease who also were given RAS inhibitor drugs.

Although the abnormalities of mesangial cells and mesangial matrix have long been considered the major components contributing to early glomerular injury in type 2 diabetes, more recent evidence has indicated that podocytes also play a critical role in the early functional and structural changes of DKD^21^. Serial investigations on kidneys from obese Zucker fa/fa rats, a model of type 2 diabetes that develops segmental glomerulosclerosis, revealed that nephropathy started with damage to podocyte, manifesting as foot process effacement and cytoplasmic accumulation of lipid droplets^22^. Early podocyte damage antedated the development of proteinuria and glomerulosclerosis in this model^23^. Similarly, in the streptozocin-induced diabetic rat model early broadening of the foot processes, initial podocyte detachment and eventually decrease in podocyte number have been reported, which were ameliorated with RAS blockade^24^. This glomerular pattern is confirmed in our type 2 diabetic patients with normo- or micro-albuminuria, in which SEM documented locally attenuated podocyte cytoplasm with occasional pseudocysts where initial cell detachment was observed. These findings are in line with our TEM observations as well as with previously TEM reports^25–28^, showing that in addition to foot process widening, the number and density of podocyte were reduced in type 2 diabetic patients even with microalbuminuria. Morphometric studies also documented that the numerical density of podocyte per glomerulus, initially reduced in patients with early diabetic nephropathy, was even further decreased in patients with overt proteinuria^26,29^, suggesting that the decreased podocyte density is a strong predictor of progressive type 2 DKD, with fewer cells predicting more rapid progression^28^.

However, the degree of GBM denudation was much more dramatic by SEM observation. Indeed, in the six diabetic patients with overt proteinuria, SEM showed almost complete acellular glomeruli with very few partly detached and phenotypically altered podocytes. In order to exclude the potential of inadvertent contamination of fixation and washing solutions, we processed the kidney samples from normoalbuminuric patients and controls with the same solutions and on the same days as those from severe DKD patients, and these did not clearly show any traces of processing alterations, ruling out the possibility of inadvertent removal of cells. Thus, these internal method controls confirmed the reliability of our processing protocol and make us confident about our observations. In addition, renal biopsy from one of our patient with overt nephropathy (Patient 4) demonstrated less severe glomerular injury by light microscopy compared with the other five patients with nephrotic range proteinuria. TEM and SEM findings were consistent with that, revealing a segmental pattern of GBM denudation, with some regions of the capillary loop surface partially covered by highly damaged podocytes. The fact that residual podocyte foot processes were clearly detectable by SEM, along with some fragments of cellular bodies, further support the hypothesis that GBM denudation in the other patients with overt nephropathy was not an artifactual issue. Notably, the presence of glomerular filtration pores in the rare preserved areas, further ruled out artifactual damage and suggested that, despite the massive structural damage clearly visible with SEM, the glomerulus was likely not globally sclerotic, but areas of the surface of the glomerular capillary tuft were still structurally intact.

The striking podocyte loss, revealed by the extensively denuded GBM, is a crucial finding which could be taken as characteristic of advanced DKD. Indeed, excellent SEM studies in the ‘70 s described a broad spectrum of glomerular diseases with overt proteinuria, and found only focal denudation of the GBM, with major alterations in podocytes, including foot process loss, microvilli, and blebs^30,31^. There is evidence that mechanical forces may have a role in podocyte detachment under physiologic and pathophysiologic conditions^32^. Thus, in DKD, long-lasting intraglomerular mechanical challenges, including glomerular hypertension, hyperfiltration, hypertrophy and outflow of filtrate from subpodocyte spaces, could promote progressive podocyte loss through detachment from the GBM. In addition, many other complex pathways contribute to the long-lasting podocyte injury and detachment in type 2 diabetes, including hyperglycemia itself, oxidative stress and inflammatory cytokines, as well as the activation of vasoactive hormonal systems^33–35^. Although SEM does not provide insight into the long-term mechanisms of podocyte injury in the diabetic setting, we hypothesize that, as a consequence of the intraglomerular hemodynamic derangement and mechanical stress as well as hyperglycemic and oxidative/inflammatory challenges persisting for years, ultimately complete loss of podocytes may occur in advanced DKD with overt proteinuria. Is this catastrophic process really ineluctable? In this regard, previous studies suggest that reversal of DKD can been achieved in humans and mice, but only rarely and under special circumstances^36^. In an experimental mouse model of DN, the leptin-deficient BTBR ob/ob mouse, it has been shown that leptin replacement resulted in near-complete reversal of both structural and functional measures of advanced DKD^37^. Immunohistochemical labeling with specific podocyte markers identified parietal epithelial cells as a possible source of new podocytes, supporting the hypothesis that podocytes may regenerate from parietal epithelial cell progenitors^29^. In our study the occasional finding of isolated podocytes, with stretched and elongated primary processes, forming bridges between the Bowman’s capsule and the GBM (Fig. 4F), could be an attempt to restore podocyte density. The relationship between the ability of parietal epithelial cells to migrate and the degree of glomerular damage, may eventually determine the even partial reversibility of podocyte loss. Therefore, given the dramatic denudation of GBM observed here by SEM, it is really difficult to imagine, at such an advanced stage of the disease, the possibility of any form of glomerular repair.

DKD is an heterogeneous condition^4^. Indeed, the evolution of the disease is often unpredictable, particularly in those patients who clearly do not follow the classic pattern of glomerular hyperfiltration progressing to persistent albuminuria associated with hypertension and declining GFR^4,38^. We acknowledge that in our study we selected a small group of patients who showed typical evolution of the disease, both in terms of natural history and correlations between laboratory parameters and histopathologic changes. In particular, our patients with advanced DKD were homogeneous, having all a proteinuria within the nephrotic range, a mild to moderate reduction of GFR and structural changes consistent with severe DKD. Furthermore, giving the impressive findings obtained by SEM, we decided to focus mainly on glomerular changes, although it is well known that also tubulo-interstitial lesions also play a pivotal role in the progression of renal impairment^17^.

Among the glomerular changes occurring in the late stages of DKD, SEM images also documented peculiar ‘crater-like’ formations on the external surface of GBM. The three-dimensional appearance of these GBM changes are reminiscent of those first described by Bonsib in 1985 with SEM in glomeruli from nephrotic syndrome cases, after the intentional removal of cellular components^39,40^. In stage I membranous nephropathy, ‘crater-like’ formations similar to those of our diabetic patients were described, though with a more diffuse distribution pattern, which are characteristic of the earliest phase of immune deposit incorporation in the GBM^40^. Usually, there are no immune complexes by immunofluorescence in glomeruli from diabetic patients. In line with that, in our patients with overt nephropathy we observed the characteristic linear homogeneous staining of IgG along glomerular and tubular basement membranes. This staining is not thought to be indicative of immune injury, but may represent stickiness of the enlarged GBM to antisera used for immunofluorescence^17^. However, immunofluorescence studies in our diabetic patients with nephrotic-range proteinuria revealed occasional granular glomerular staining for IgM and C3, in addition to the aforementioned linear IgG. Therefore, we may consider ‘crater-like’ formations on the GBM surface detected by SEM as equivalent of the immune deposits composed of IgM and C3. Of interest, Haas described subepithelial immune deposits of incidental healed post-infectious glomerulonephritis superimposed on diabetic nephropathy^41^. In addition to ‘crater-like’ formations, ‘tunnels and cavities’ were also part of the ultrastructural GBM defects. As the diameters of most of these tunnels and cavities were far larger than albumin molecules, it is hypothesized that these enlarged structures allow serum protein to pass through the GBM from the capillary lumen to the urinary space, causing massive proteinuria, as seen in our patients.

In conclusion, SEM findings indicate that in the later stage of proteinuric DKD glomerular capillary barrier is completely subverted in its cellular and matrix components with possible implications for disease treatment. Indeed in this setting any conventional and novel drugs would fail to affect renal disease progression^13,14,42^, since no cellular or matrix targets for their local pharmacodynamic effect are available^43^. This observation should persuade physicians to start potentially renoprotective therapies at the very early phase of diabetes when the integrity of glomerular cellular components is largely preserved, in order to prevent later untreatable glomerular injury. In the future earlier intervention clinical trials with more specific therapies currently in the pipeline for treating DKD should be designed.

More in general, the present study indicates that, although SEM is mainly a great research tool, it could also provide clinicians with more insightful information that would complement the clinical pathology diagnostic approach provided by TEM.

## Materials and Methods

### Patient characteristics

Computerized records from the Unit of Nephrology at Azienda Socio-Sanitaria Territoriale Papa Giovanni XXIII (Bergamo, Italy) were reviewed to identify all renal biopsy specimens from six patients with diabetic nephropathy and overt proteinuria. We also identified two autopsy cases of type 2 diabetic patients with early stage of diabetic nephropathy (normoalbuminuria, microalbuminuria) in the Unit of Pathology at the same institution. Patients’ medical records were reviewed for demographics, clinical features of diabetic nephropathy, medication history, parameters of renal function, treatment, and outcome.

A 61-year-old woman with a history of type 2 diabetes since 1994 and obesity, on oral antidiabetic therapy (metformin 1 gr. t.i.d, glimepiride 2 mg b.id.), good blood pressure control with two antihypertensive agents including the ARB Valsartan (80 mg/d), died suddenly for cardiovascular event and an autopsy was performed with tissue sampling 24 hours later. The most recent available laboratory test reported normal renal function (s. creat. 0.8 mg/dl), fasting blood glucose 152 mg/dl, and normoalbuminuria (5 µg/min).

A 70-year-old man with type 2 diabetes since 1998, on oral antidiabetic treatment with metformin (400 mg/die), glibenclamide (5 mg t.i.d), with a history of smoking and hypertension, developed microalbuminuria (102 µg/min) beginning in 2012. At that time, he was on a low dose of ACEi ramipril (2.5 mg/day), then increased to 5 mg/day, which lowered urinary albumin excretion (UAE). A few years later, he died suddenly for a cerebrovascular event and an autopsy was performed with tissue sampling 24 hours after death. At the most recent clinical evaluation UAE was still in the microalbuminuric range (53.5 µg/min), with normal renal function (s.creat. 0.96 mg/dl), fasting blood glucose was 174 mg/dl and the patient was given two antihypertensives to control blood pressure. Four male patients (aged 60 to 73) with a history of type 2 diabetes were referred to our Nephrology Unit due to nephrotic proteinuria, and underwent a renal biopsy between 2011 and 2016 (Supplementary Table S1). They all had mild to moderate renal insufficiency (eGFR, 50, 71, 84, 76 ml/min/1.73 m^2^). All of them had near normal fasting blood glucose on oral antidiabetic agents, and good blood pressure control (130-140/70–80 mmHg) with at least two antihypertensive agents, including ACEi or ARBs. Additionally, two female diabetic patients (aged 50 and 55) underwent a renal biopsy in the beginning of 2017 due to overt proteinuria (Supplementary Table S1). They had moderate to severe renal dysfunction (eGFR, 22, 35 ml/min/1.73 m^2^). Patient 5 had a longstanding insulin-dependent type 2 diabetes with poor glycaemic control (glycated haemoglobin of 101 mmol/mol) and uncontrolled hypertension (150–160/80–90 mmHg). At the time of renal biopsy she was taking a calcium antagonist and an ACEi, which was discontinued due to iperkalemia. Patient 6 was diagnosed with type 2 diabetes in 2014, but she already had severe micro- and macro-vascular complications (retinopathy and multiple diabetic foot ulcers). At the time of renal biopsy she was on a single oral antidiabetic agent (metformin 1 gr. t.i.d) with good glycaemic control (glycated haemoglobin of 43 mmol/mol) and she was receiving dual blockade of the renin-angiotensin-aldosterone system. In addition, two non-diabetic controls undergoing nephrectomy for kidney adenocarcinoma without histological evidence of glomerular disease and no history of renal disease, were included. All experimental protocols involving human subjects are carried out in accordance with the Declaration of Helsinki and good clinical guidelines, and approved by the Ethical Committee of the Azienda Socio Sanitaria Territoriale (ASST) Papa Giovanni XXIII. Written informed consent was obtained from patients enrolled in the study.

### Renal histology

Routine processing pipelines for kidney biopsies were utilized for specimen preparation. For light microscopy, formalin-fixed, paraffin-embedded kidney biopsy tissue was cut at 2-μm thickness, deparaffinized and stained with hematoxylin and eosin, periodic acid–Schiff reagent and trichrome.

### Transmission Electron Microscopy (TEM) analysis

Biopsy specimens were immersed in ice-cold 2.5% glutaraldehyde in 0.1 M cacodylate buffer (pH 7.4) immediately after their extraction and sectioning, and fixed for 4 h at 4 °C, carefully handling samples to avoid *ex vivo* artefacts. After washing in cacodylate buffer, kidney fragments were then postfixed in 1% osmium tetroxide for 1 h, dehydrated through ascending grades of alcohol, and embedded in Epon resin (Electron Microscopy Science, Hatfield, PA). Ultrathin sections (60 to 100 nm) were cut on an EM UC7 ultramicrotome (Leica Microsystems, Mannheim, Germany), stained with uranyl acetate and lead citrate, and examined with TEM (Morgagni 268D, Philips, Brno, Czech Republic).

### Scanning Electron Microscopy (SEM) analysis

For SEM analysis, as for TEM samples, all the analyzed kidney biopsies were immersed in ice-cold 2.5% glutaraldehyde (buffered with 0.1 M sodium cacodylate buffer, pH 7.4) immediately after their extraction and sectioning, and fixed for 4 h at 4 °C, carefully handling samples to avoid *ex vivo* artefacts^15^. SEM analysis was performed on the remaining tissue (usually 1 × 2 mm) of the fixed biopsy sample used for TEM diagnosis. Sections were repeatedly washed in cacodylate buffer and postfixed in 1% osmium tetroxide for 1 hour. Fixed specimens were dehydrated through increasing concentrations of ethanol, starting with 10, 50, 70 and 90% of ethanol. Once the tissues were equilibrated in anhydrous 100% ethanol, they were rinsed with liquid carbon dioxide with a Bal-Tec 030 critical point dryer (BAL-TEC AG, Balzers, Liechtenstein). Kidney specimens from non-diabetic controls, serving as method control, were processed at the same time and with same solutions used for samples from normoalbuminuric, microalbuminuric and severe DKD patients. Samples were mounted on stubs, and coated with a thin layer of atomic gold particles in a sputter coater (Agar Scientific, Stansted, UK). Coated specimens were observed through SEM using secondary electron detection (Supra 55, Zeiss, Oberkochen, Germany). Acceleration voltage was set to 0.4 to 1.5 kV, working distance to 4 to 6 mm and enlargement up to 20 kx.

### Data availability

All data generated or analysed during this study are included in this published article (and its Supplementary Information files).

## Electronic supplementary material

Supplementary Information
