# Supplementary material for: Early and late scanning electron microscopy findings in diabetic kidney disease
Source: Sci Rep. 2018 Mar 20;8:4909. doi: 10.1038/s41598-018-23244-2 (PMC5861033; doi:10.1038/s41598-018-23244-2)
Supplement: Supplementary file 1 — Supplementary Information [file 41598_2018_23244_MOESM1_ESM.pdf]

SUPPLEMENTARY INFORMATION

Early and late scanning electron microscopy findings in diabetic kidney disease

Sara Conti[1], Norberto Perico[1], Rubina Novelli[1], Camillo Carrara[1],

Ariela Benigni[1],Giuseppe Remuzzi[1,2,3*]

[1]IRCCS - Istituto di Ricerche Farmacologiche Mario Negri, Bergamo, Italy

[2]Unit of Nephrology and Dialysis, Azienda Socio-Sanitaria Territoriale Papa Giovanni XXIII, Bergamo, Italy

[3]Department of Biomedical and Clinical Sciences, 'L. Sacco', University of Milan, Milan, Italy

Supplementary Table S1: demographic and clinical characteristics of six patients with type 2 diabetes and overt nephropathy at the time of kidney biopsy.

**Patient**	**Gender**	**Age** (years)	**UPE** (g/24h)	**eGFR *** (ml/min/1.73 m^2)	**ACEi/ARBs**
1	M	73	4.36	50	Yes
2	M	60	4.14	71	Yes
3	M	64	3.53	84	Yes
4	M	63	4.25	76	Yes
5	F	50	5.60	22	Yes
6	F	55	15.30	35	Yes

* eGFR by CKD-EPI formula; M: male; UPE: urinary protein excretion.

Supplementary Figure Legends

Figure S1

Glomerular ultrastructural architecture from SEM of control non-diabetic patients. (A-C) Scanning electron micrographs with the corresponding high magnification insets (B-D) show well-preserved glomeruli with normal peripheral capillary loop ultrastructure. (B-D) The outer surfaces of glomerular capillaries are covered by highly branched podocytes revealing a perfectly normal interdigitating foot process pattern.

Figure S2

Low power view of renal cortical parenchyma from Patient 3 showing typical diffuse nodular glomerulosclerosis of advanced diabetic nephropathy. The surrounding tubular parenchyma reveals diffuse interstitial fibrosis and focal tubular atrophy (Periodic acid-Schiff).

Figure S3

Glomerular ultrastructural architecture from Patient 4. (A) Low power view of renal cortical parenchyma observed by SEM. (B) A large glomerulus protrudes from the renal cortex surface. (C-E) SEM findings reveal a segmental pattern of GBM denudation, with a few regions of the capillary loop surface covered by nearly preserved foot processes (arrowhead). Rare preserved areas were clearly detectable by SEM, along with highly damaged areas with denuded GBM (arrow), connected by a microvillous podocyte (asterisk). (F-G) Occasional filtration pores are noted between two adjacent foot processes (insets), in the rare preserved areas of the glomerular tuft.

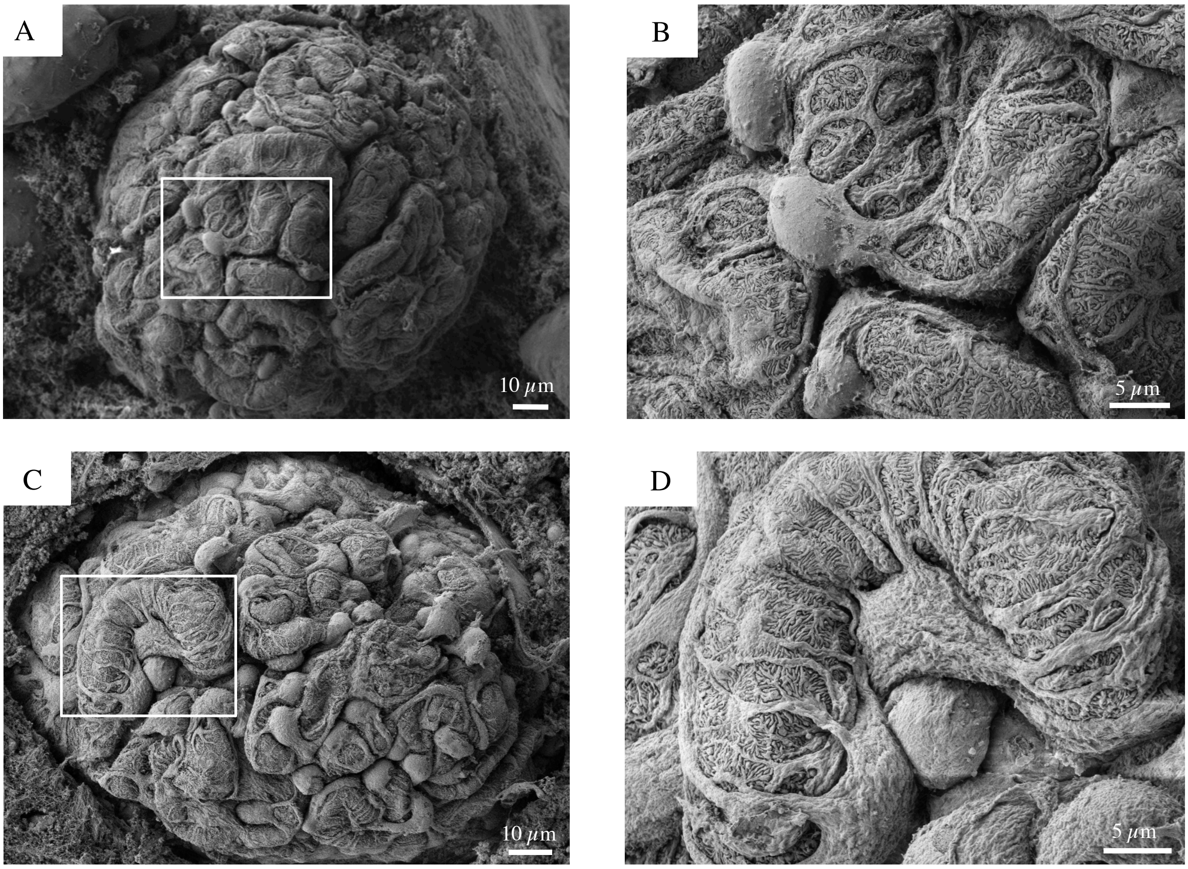

Supplementary Figure S1

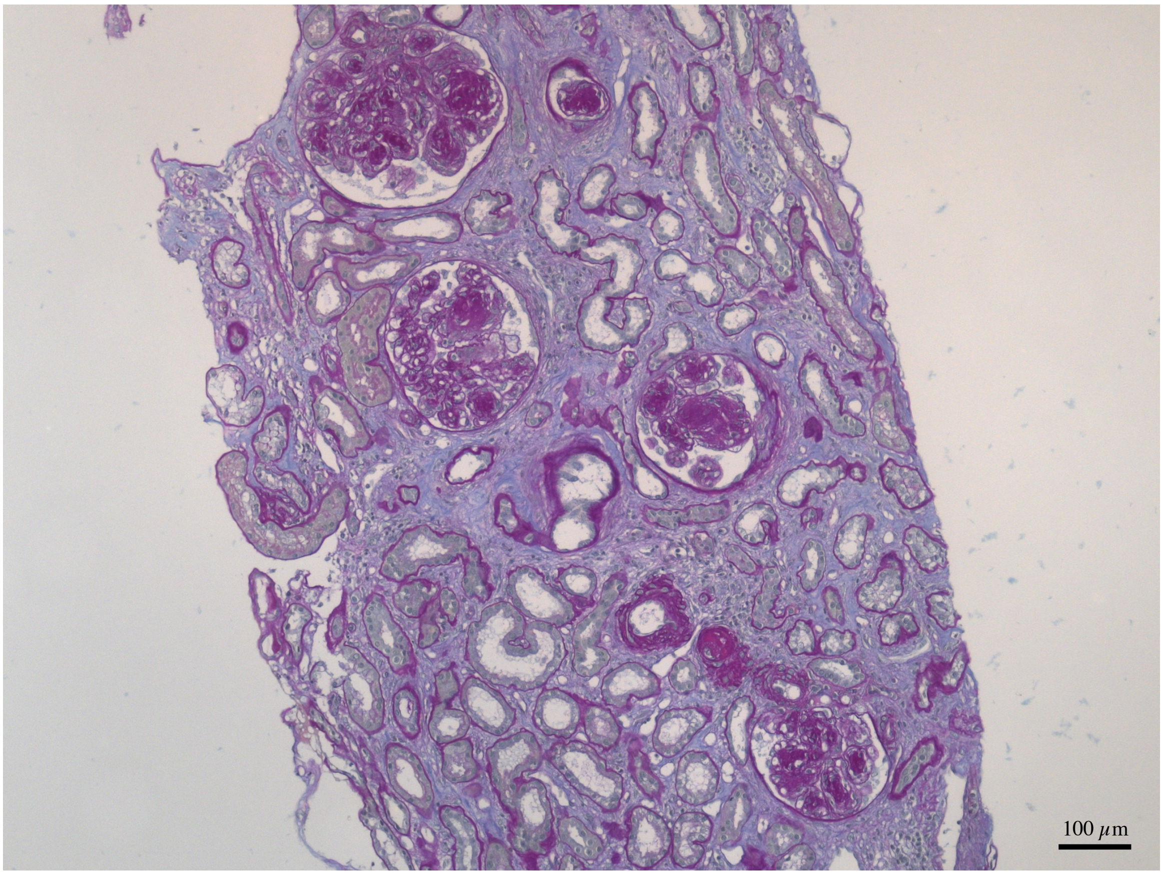

Supplementary Figure S2

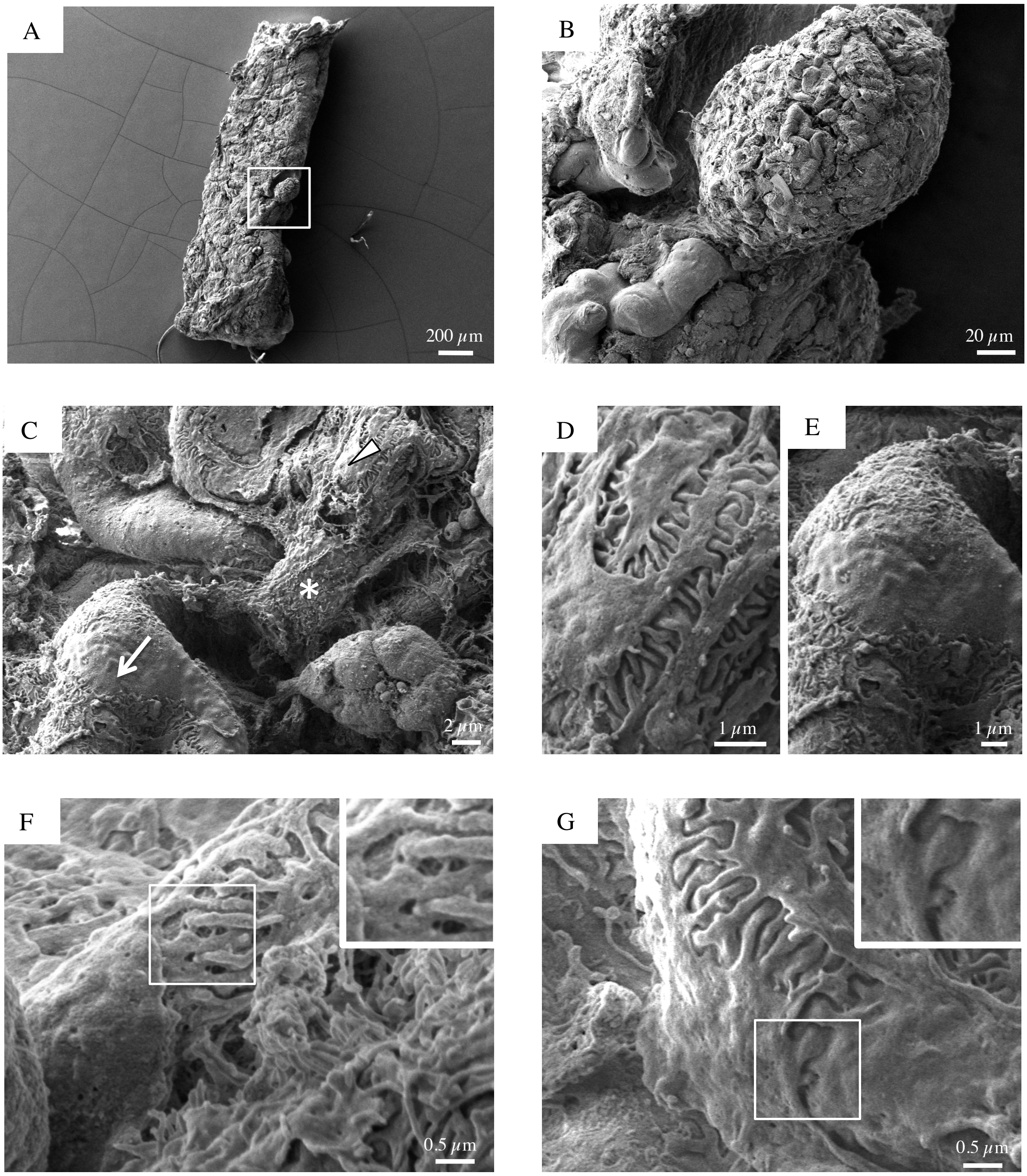

Supplementary Figure S3
